# Supplementary material for: A modelling assessment of the impact of control measures on highly pathogenic avian influenza transmission in poultry in Great Britain
Source: PLoS Comput Biol. 2026 Jan 5;22(1):e1013874. doi: 10.1371/journal.pcbi.1013874 (PMC12810912; doi:10.1371/journal.pcbi.1013874)
Supplement: S1 Text — A description of the data used alongside details of the model assumptions and outputs from the model fitting process. (PDF) [file pcbi.1013874.s001.pdf]

Supplementary Material: A modelling assessment of the impact of control measures on highly pathogenic avian influenza transmission in poultry in Great Britain

Christopher N Davis[1,2,3], Edward M Hill[4,5], Chris P Jewell[6], Kristyna Rysava[7], Robin N Thompson[7], and Michael J Tildesley[1,2,3]

[1]Zeeman Institute for Systems Biology and Infectious Disease Epidemiology Research (SBIDER), University of Warwick, Coventry, UK
[2]Mathematics Institute, University of Warwick, Coventry, UK
[3]School of Life Sciences, University of Warwick, Coventry, UK
[4]Civic Health Innovation Labs and Institute of Population Health, University of Liverpool, Liverpool, UK
[5]NIHR Health Protection Research Unit in Emerging and Zoonotic Infections, University of Liverpool, Liverpool, UK
[6]Department of Mathematics and Statistics, Lancaster University, Lancaster, UK
[7]Mathematical Institute, University of Oxford, Oxford, United Kingdom

* Corresponding author: c.davis.7@warwick.ac.uk

Data

The demographic data on poultry premises includes the centroid of each premises polygon (defined as a CPH — County/Parish/Holding number — entity). The poultry case data provides a latitude and longitude value of the case. To match the correct premises from the demographic data to the reported IP in the case data, we found the closest premises in the demographic data to the case coordinates with similar poultry populations between the two data sets. Specifically, we consider all premises within a 2 km radius of the case data coordinates and match the closest premises with the same bird species types and the same size of premises in terms of number of birds (less than 50, from 50 to 1,000 or more than 1,000). If no match is found to all the criteria, we match the remaining premises if there is a valid premises in the demography data in very close proximity (less than 200 m) or if the bird type does not match, but the numbers of birds do match, and vice versa. Premises with still no match are added to the demographic data set. Of the 200 premises in our data set, 158 were matched in the first step, a further 6 were matched by close proximity, and another 6 were matched by number of birds or bird type. The final 30 premises were added to the data set.

Our distribution of poultry premises sizes is given in a histogram (Figure A-(A)). There are many small-sized premises (less than 100 birds) with a decreasing number of premises with larger sizes. However, there is a second small peak of premises with a large number of birds (approximately 20,000 birds). Spatially, most poultry is kept in rural areas of Great Britain with a relatively high density in the East of England, South West and close to the border with Wales in the West Midlands (Figure A-(B)). The most common poultry type kept are Galliformes (Figure A-(C)) with approximately 282 million, compared to 4 million waterfowl and 43 million other birds. The most common premises type is Galliformes only, followed by mixed, other birds only, and then waterfowl only, with many fewer premises (Figure A-(L)). The distributions of birds in premises of particular types shows that, similarly to the total number of birds, Galliformes only and waterfowl only premises are bimodal in size, with birds kept in small or large numbers with two peaks in the distribution (Figure A-(D) and -(E)). Other birds only premises commonly have moderate numbers of kept birds (Figure A-(F)), and mixed species type premises have a similar distribution to all premises types combined (Figure A-(G)). Spatial distributions of total bird species type numbers are shown in Figure A-(I–K).

For considering spatial strategies, when we use enhanced biosecurity in the main text, we use different spatial

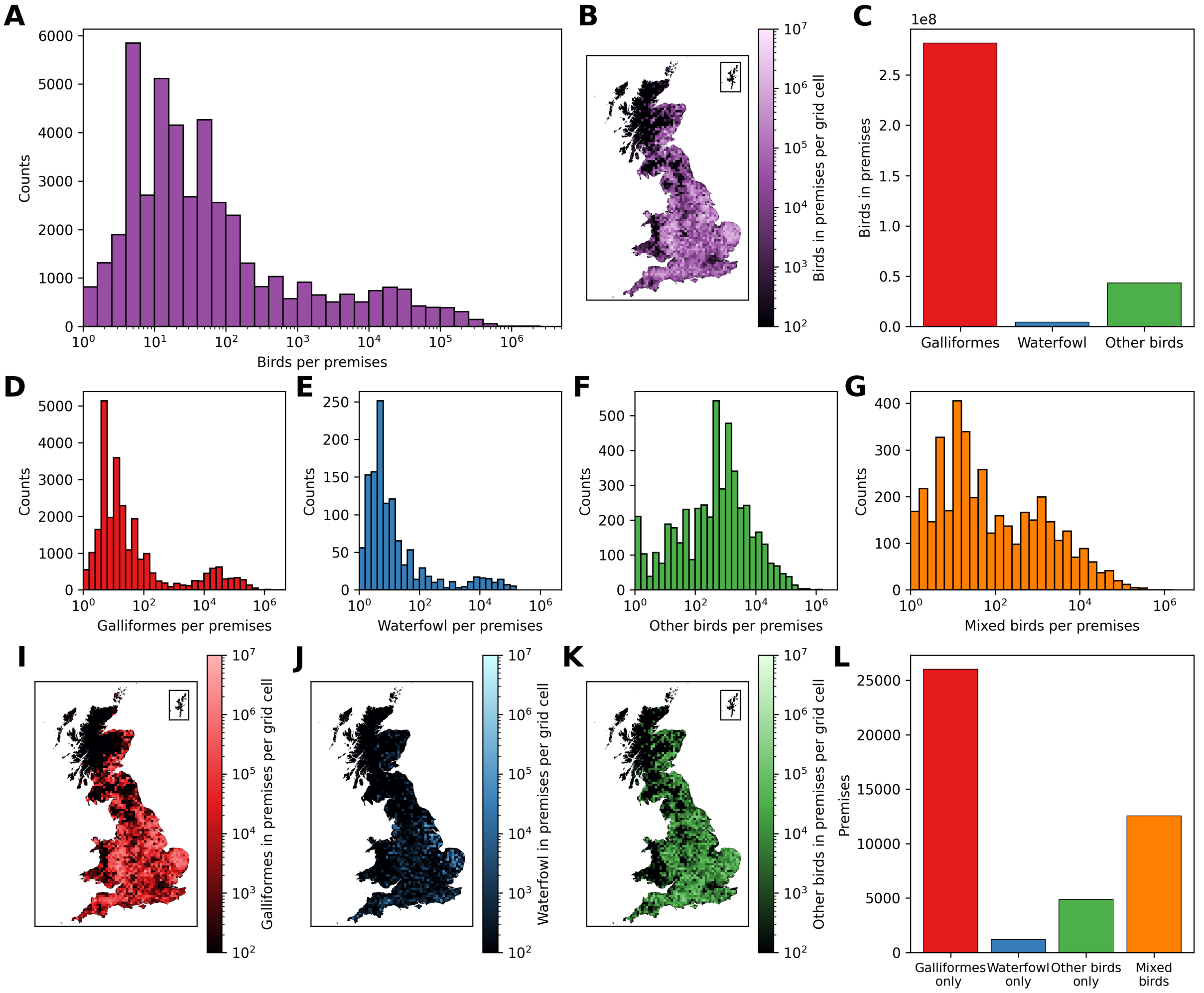

Fig A. Summary of poultry premises data. (A) Distribution of the total number of birds on each poultry premises in the data set. The x-axis is shown on a logarithmic scale. (B) Spatial distribution for the total number of birds within all premises within a 10 km by 10 km grid cell. (C) The total number of birds for each bird type across all poultry premises. (D–G) Distribution of the number of birds divided by premises with: (D) Galliformes only, (E) waterfowl only, (F) other birds only and (G) mixed species premises. A logarithmic scale is used for the x-axis. (I–K) Spatial distribution for the total number of birds of each species within premises within a 10 km by 10 km grid cell for: (I) Galliformes, (J) waterfowl and (K) other birds. (L) Number of premises for each species type: Galliformes only, waterfowl only, other birds only and mixed birds. Source of map boundaries: Office for National Statistics licensed under the Open Government Licence v.3.0. The shapefiles used can be found at `https://geoportal.statistics.gov.uk/datasets/5a393192a58a4e50baf87eb4d64ca828_0/explore`.

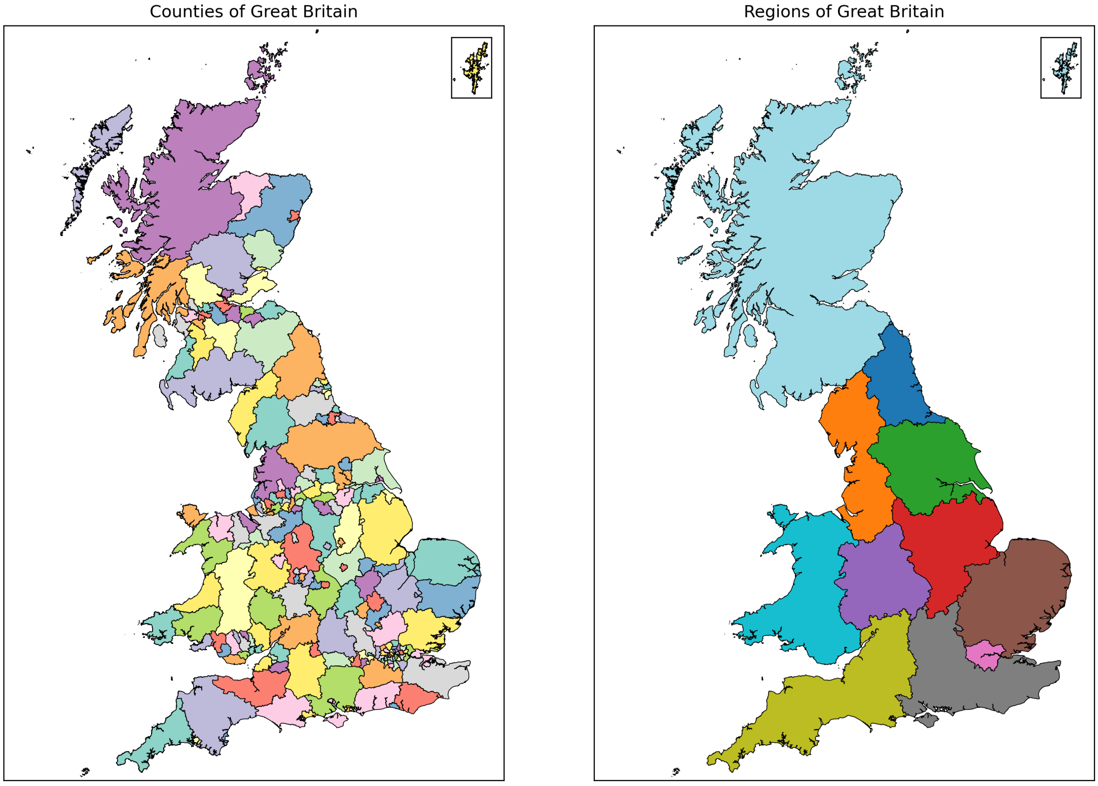

Fig B. Counties and regions of Great Britain. Boundaries of the counties and regions are shown on a map. Source: Office for National Statistics licensed under the Open Government Licence v.3.0. The shapefiles used can be found at `https://www.arcgis.com/home/item.html?id=2cc79b94571a438bb05bc9ec16831529` and `https://geoportal.statistics.gov.uk/datasets/5a393192a58a4e50baf87eb4d64ca828_0/explore` respectively.

scales, including at the county and region levels. These are all areas of different shapes and sizes and are displayed in Figure B. There are 207 counties included and 11 regions in Great Britain.

Model fitting

We assume the infection events occurred according to a continuous-time non-homogeneous Poisson process, given infection rate $\lambda_j(t)$. We use this method for computational efficiency in exploring the state space, noting that the approximation from continuous time inference to discrete time simulation will provide negligible differences. The time to notification from being infectious $(N_i - I_i)$, is also assumed to be gamma distributed. Therefore, the joint posterior of the parameters can be described by:

$$\pi(\theta \mid N) \propto \left(\prod_{i=1}^{n_I} \lambda_i(E_i)\right)\left(\exp\left(-\sum_{t=1}^{T}\sum_{i=1}^{n} \lambda_i(t)\right)\right)\left(\prod_{i=1}^{n_I} (N_i - I_i)^{a-1} \exp\left(-b(N_i - I_i)\right)\right)\left(\prod_{p=1}^{|\theta|} f_p(\theta_p)\right), \quad (1)$$

where n is the total number of premises and n_I is the total number of these premises that are infected up to time T. The prior distributions of the fitted model parameters are given by $f_p((\theta_p)$ for parameters θ_p, where $\boldsymbol{\theta} = \{\epsilon_0, \gamma_0, \gamma_1, \delta, \psi_0, \psi_1, \psi_2, \phi_0, \phi_1, \phi_2, \xi_1, \xi_2, \zeta_1, \zeta_2, \nu_0, \nu_1\}$. In practice, we fit log-transformed parameters and account for this in the MCMC accordingly.

We use a reversible Markov chain Monte Carlo (MCMC) method, which proposes a new set of parameters in each MCMC iteration and then proposes a fixed number of additional events for uniformly chosen random premises, which are:

i) Update the time between a premises becoming infectious and being notified,

ii) Add an occult infection as a new infection that has not yet been detected at the end of the simulation period,

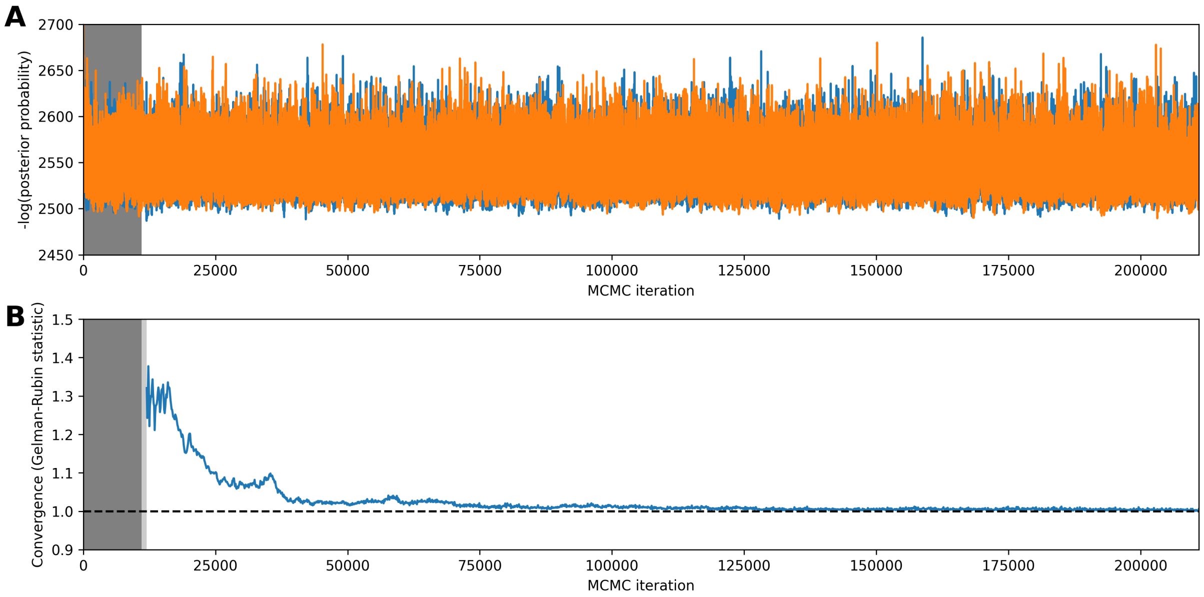

Fig C. MCMC chain and convergence. (A) The two MCMC chains showing the negative logarithm of the posterior probability by iteration. A burn-in of 11,000 iterations is used (shown by the dark grey background), followed by a further 200,000 iterations. (B) Convergence of the two chains by MCMC iteration as calculated by using the Gelman-Rubin statistic on 1000 thinned samples. The dashed line at 1 shows the chains converging towards this value as the MCMC process is simulated. The dark grey region shows the neglected burn-in period, and the lighter grey region shows the period before 1,000 values post-burn-in are available to calculate the statistic.

iii) Remove a previously added occult infection.

We choose the fixed number of additional events to be equal to 5% of the total number of infected premises in the data set. Therefore, we include ten possible additional events per MCMC iteration in the fitting process.

We perform 211,000 MCMC iterations for two independent chains, which for the first 10,000 we update each parameter separately in order to get a better initial starting point before updating all parameter values as a set simultaneously for the following iterations (Figure CA). For obtaining posterior distributions, we remove these first 10,000 iterations and an additional 1,000 of the subsequent iterations as a burn-in period, leaving 200,000 iterations from which we obtain posterior estimates.

These chains visually appear to have converged (Figure CA), however, we also calculate the convergence of these chains using the Gelman–Rubin statistic [1] (Figure CB). This confirms that as we increase the number of MCMC iterations, the Gelman–Rubin statistic trends towards a value of 1, and so we have likely performed a sufficient number of iterations.

For the posterior parameter sets, we take the 200,000 MCMC iterations and thin these to reduce the auto-correlation and get a set of 1,000 parameter sets for each chain for a total of 2,000 posterior parameter sets. We observe that the posterior distributions for all parameters differ from their priors while remaining smooth and well-constrained, indicating that the data have informed the parameter distributions and that the fitting process converged successfully (Figure D). Fitted parameter values are given in Table A.

Similarly, we output a unique time to notification posterior distribution for each of the infected premises. We show an example of three premises of the 200, which all exhibit similar features, alongside the distribution of the average time to notification time across all the premises (Figure E). We see that during the fitting process, the algorithm is generally selecting for a shorter time to notification than given by the priors, with a mean value of approximately 6.7 days before notification.

The number of occult infections remains low, with the posterior only including between zero and ten additional premises, with two being the most likely value (Figure F). This is to be expected since the end of the simulations is at the end of the summer (30 September 2023), where infections are typically at a low number before migratory wild birds arrive in Great Britain. We show the probability that each premises is included as an occult infection, where the largest probability is 0.0012, demonstrating that all premises are much more likely to be not infected. The premises with the highest probability of being occult is the closest premises to the last

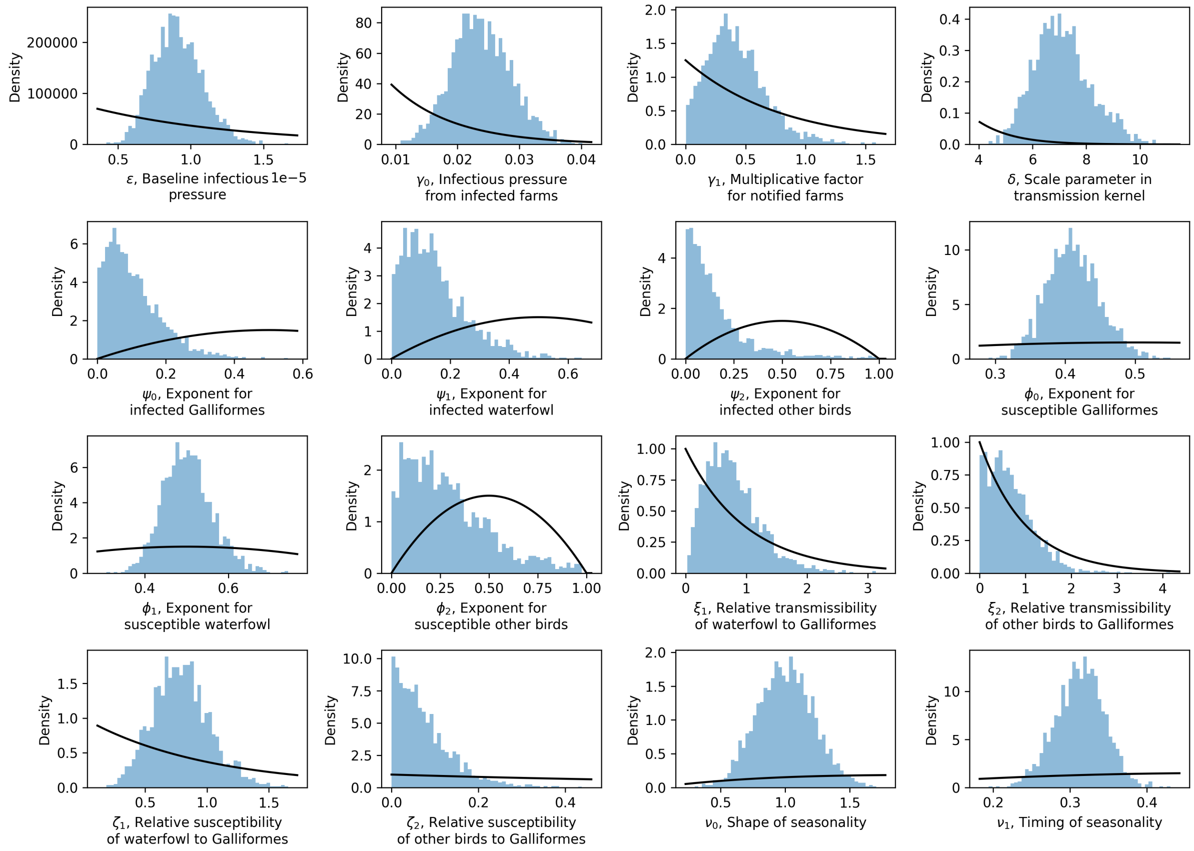

Fig D. Posterior parameter distributions. Parameter distributions for the 16 model parameters after Markov chain Monte Carlo. The black lines show the prior distributions used.

Parameter	Mean value	95% confidence interval
ϵ	0.00000911	[0.00000617, 0.0000127]
γ_0	0.0239	[0.0150, 0.0337]
γ_1	0.408	[0.0452, 1.02]
δ	7.11	[5.25, 9.40]
ψ_0	0.106	[0.00554, 0.310]
ψ_1	0.148	[0.00935, 0.409]
ψ_2	0.164	[0.00602, 0.687]
ϕ_0	0.411	[0.339, 0.490]
ϕ_1	0.503	[0.396, 0.631]
ϕ_2	0.288	[0.0172, 0.813]
ξ_1	0.782	[0.137, 1.89]
ξ_2	0.687	[0.0212, 1.87]
ζ_1	0.793	[0.377, 1.28]
ζ_2	0.0762	[0.00223, 0.243]
ν_0	1.00	[0.593, 1.44]
ν_1	0.313	[0.248, 0.371]

Table A. The mean value of parameter estimates from the posterior distributions presented with 95% confidence intervals. All values are presented to three significant figures

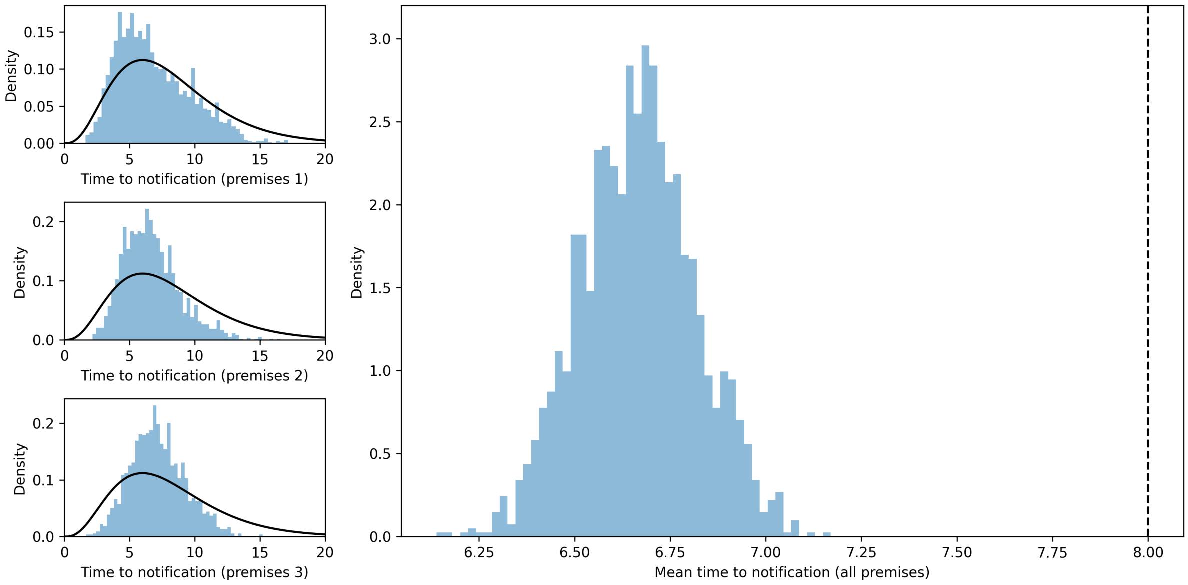

Fig E. Distributions of the time to notification. Time to notification distributions for three example premises of the 200 infected premises in the 2022–23 season. The black lines show the prior distribution, which is the same for each premises. To the right is the distribution of the mean value across the 200 infected premises, where the dashed line is the mean of the prior distribution.

recorded infection (on 26 September 2023), indicating that the model results are consistent with the data. Indeed, using data since September 2023, we truly observed zero IPs for the time scale of occult infections on 30 September 2023, in agreement with the modelled results.

Marked on the map in Figure F are red crosses and dates, which correspond to the infected premises in the last month of the data period. There are no premises with large probabilities close to the infected premises other than the premises infected on 26 September, as they are not sufficiently close to the end of the modelled period compared to the incubation period plus the time to notification. The other premises with relatively large probabilities of being occult infections (albeit still less than 0.001) are typically premises with large numbers of kept birds.

Model assumptions

Throughout the main text, we assumed the transmission kernel takes a Cauchy form (Main Text, Equation 4). Here, we also considered an exponential form given by:

$$K_{ij} = K\left(d_{ij}\right) = A \exp\left(-\delta d_{ij}\right), \tag{2}$$

where A is a constant such that the median value of γ_0 remains unchanged. The parameter distributions after MCMC are shown in Figure G.

The posterior parameter estimates remain broadly unchanged, with the exception of the δ parameter, which is shown in separate panels due to different scales. This is different because the parameterisation is inherently different due to the differing functional form of the kernel. However, the overall shape of the transmission kernels are similar (Figure H).

The mean exponential kernel is smaller at close distances, is larger for moderate distances and smaller again for very large distances. This is despite there being a strong overlap in the uncertainty. Due to the minimal difference, we chose to use the Cauchy form of the kernel in the main text because of the higher transmission at closer distances.

Seasonality is included in the model within the background infection term:

$$\epsilon(t) = \epsilon_0 \exp\left(-\nu_0 \left(1 + \cos\left(2\pi \left(\frac{t}{365} - \nu_1\right)\right)\right)\right), \tag{3}$$

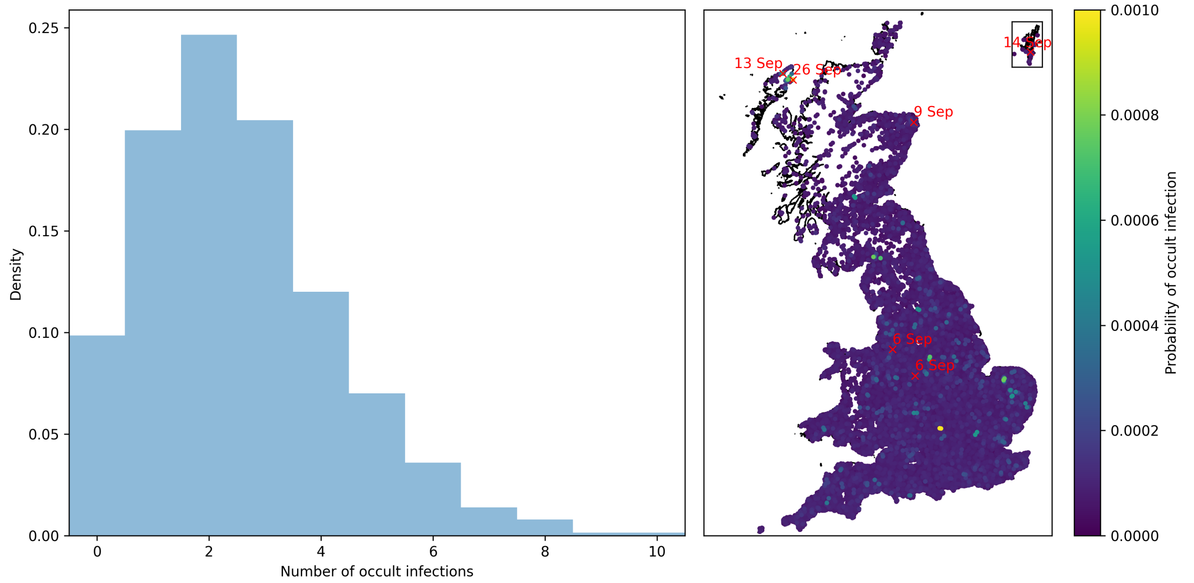

Fig F. Occult infections. Distribution for the number of occult infections and a map of the probability that each premises is an occult infection. Additionally marked in red are the true infected premises in the last month of the season, with dates of notification shown. Source of map boundaries: Office for National Statistics licensed under the Open Government Licence v.3.0. The shapefiles used can be found at `https://geoportal.statistics.gov.uk/datasets/5a393192a58a4e50baf87eb4d64ca828_0/explore`.

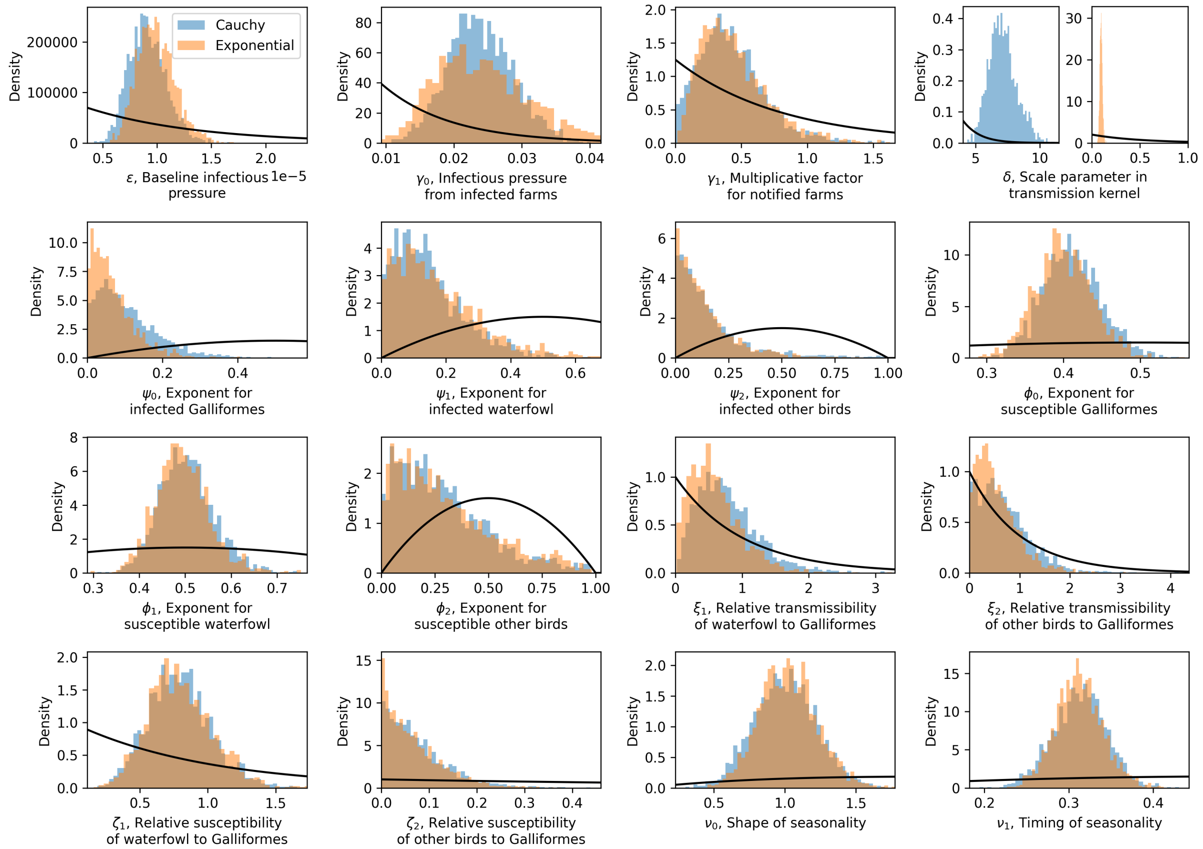

Fig G. Posterior parameter distributions for models with Cauchy and exponential transmission kernels. Parameter distributions for the 16 model parameters after Markov chain Monte Carlo. The Cauchy kernel of the main text is shown in blue, and the exponential kernel is shown in orange. The black lines show the prior distributions used.

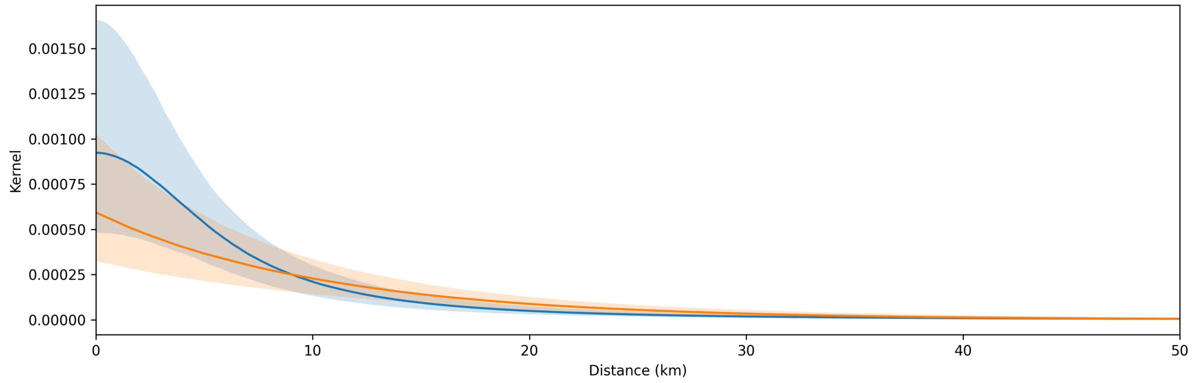

Fig H. Transmission kernels. The fitted functional form of the transmission kernels is shown with 95% prediction intervals using the posterior parameter distributions. The Cauchy kernel of the main text is shown in blue, and the exponential kernel is shown in orange.

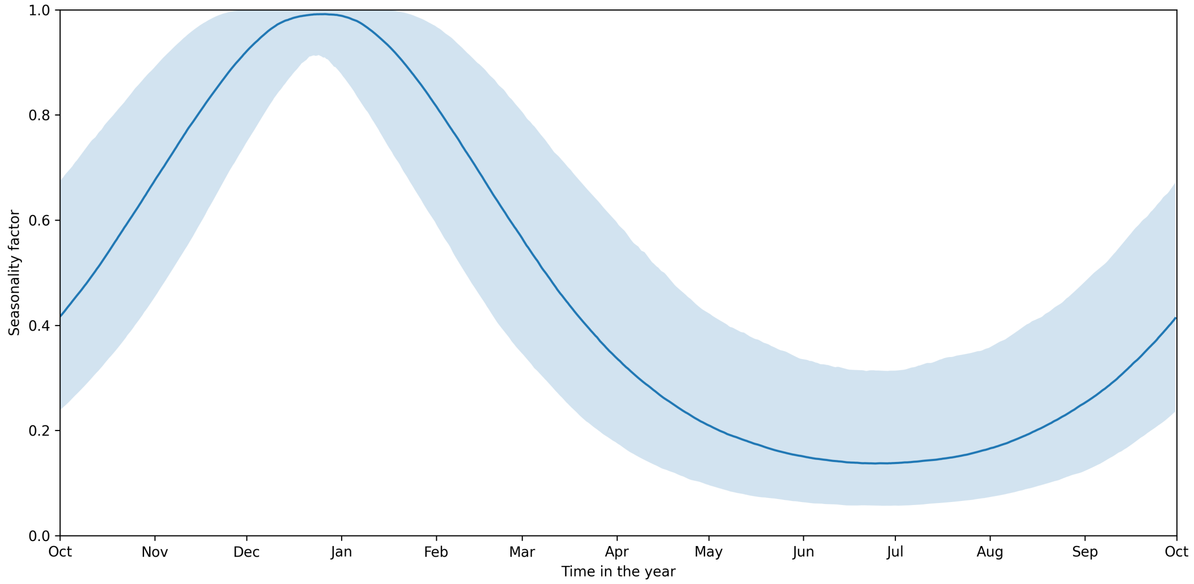

Fig I. Seasonality of background infections. The fitted functional form of the seasonality factor is shown with 95% prediction intervals using the posterior parameter distributions. The seasonality factor is an equation that multiplies with the fitted parameter ϵ_0 to give $\epsilon(t)$.

In the fitted model, we see that the posterior parameter distributions give a seasonality relatively sharply across November to February and is likely to reach its maximum in late December. Through much of the spring and summer, transmission is much reduced with the seasonality factor close to 0.2 (Figure I).

Model validation

Complementing Figure 2 in the main manuscript, we show the same results, but for the number of birds on each of the IPs (Figure J). We similarly see a good match to the temporal data in our model simulations and the spatial fit is similarly well matched with most data points for the proportion of birds infected falling within 95% prediction intervals. Specifically, the infected premises in the East of England are larger premises, meaning the model fit now is within the prediction intervals. Infected birds in Scotland are similarly underestimated, as in Figure 2, and infected birds in the South East are also slightly underestimated in the model.

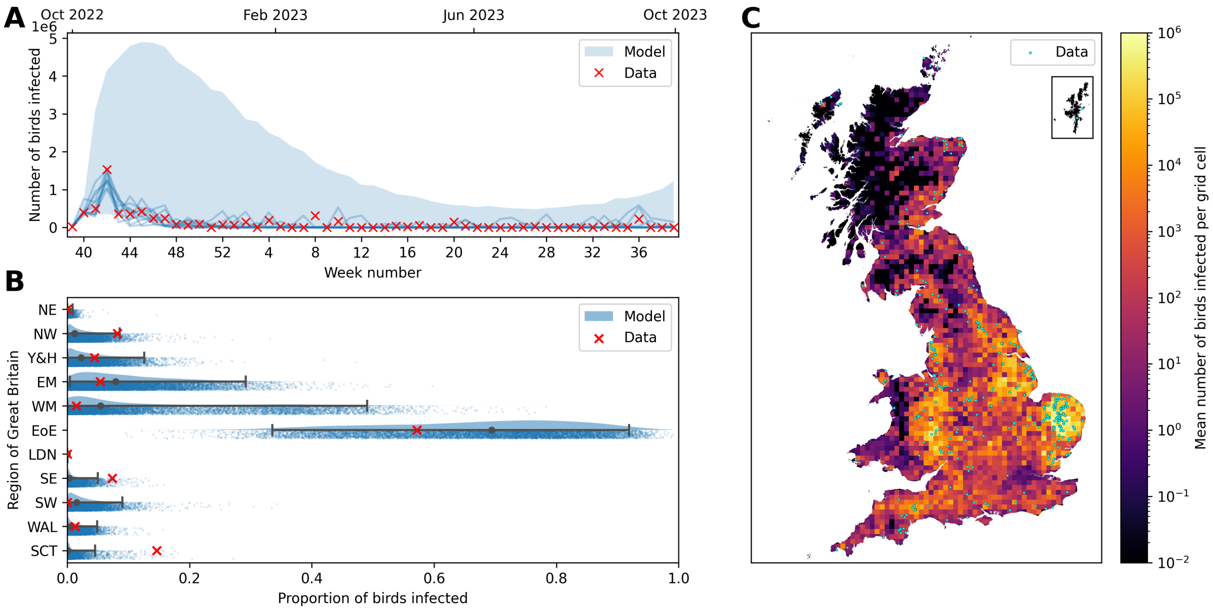

Fig J. Comparison of model simulations to infected poultry on premises for 1 October 2022 – 30 September 2023. (A) Time series of the weekly number of poultry on infected premises (IPs). The red crosses indicate the data, while the shaded blue regions show the 95% prediction intervals of stochastic model simulations. Blue lines indicate the best fitting 10 individual realisations of the simulation out of the total 10,000. (B) Raincloud plots [2] for the proportion of poultry on IPs across the eleven geographical regions of Great Britain. The data are represented by red crosses, while the central prediction interval and rainclouds show model simulations. The grey dot shows the median value, and the whiskers give the full 95% prediction interval. Above each interval is a half-violin plot of the distribution of the simulations, and below is a jittered scatter of each individual simulation. The names of the regions in full are: NE – North East, NW – North West, Y&H – Yorkshire and the Humber, EM – East Midlands, WM – West Midlands, EoE – East of England, LDN – London, SE – South East, SW – South West, WAL – Wales, SCT – Scotland. (C) Map of Great Britain divided into 10 km × 10 km grid cells coloured to show the mean number of poultry birds on IPs in each cell for the 2022–23 season. Blue dots overlaying the grid cells show the locations of the true IPs in this season. Source of map boundaries: Office for National Statistics licensed under the Open Government Licence v.3.0. The shapefiles used can be found at `https://geoportal.statistics.gov.uk/datasets/5a393192a58a4e50baf87eb4d64ca828_0/explore`.

References

[1] Andrew Gelman and Donald B Rubin. Inference from iterative simulation using multiple sequences. *Statistical science*, 7(4):457–472, 1992.

[2] Micah Allen, Davide Poggiali, Kirstie Whitaker, Tom Rhys Marshall, Jordy van Langen, and Rogier A Kievit. Raincloud plots: a multi-platform tool for robust data visualization. *Wellcome open research*, 4:63, 2021.
