# Supplementary material for: A modelling assessment of the impact of control measures on highly pathogenic avian influenza transmission in poultry in Great Britain
Source: PLoS Comput Biol. 2026 Jan 5;22(1):e1013874. doi: 10.1371/journal.pcbi.1013874 (PMC12810912; doi:10.1371/journal.pcbi.1013874)
Supplement: Attachment — Submitted filename: reviewer_response.pdf [file pcbi.1013874.s002.pdf]

Dear Prof. Blyuss and Prof. Flegg,

Thank you for the opportunity to respond to the comments of the reviewers and revise our manuscript based on their feedback. The reviewers' comments have enabled us to improve the clarity of the work, and the addition of the requested figures and tables further supports our analysis. We believe that this process has greatly improved the quality of our manuscript, and we hope that it is now acceptable for publication in PLOS Computational Biology.

A full response to the reviewers' comments is detailed below.

Yours sincerely,

Christopher Davis

University of Warwick

(on behalf of all co-authors)

Reviewer #1:

General comments

**Reviewer**	**Response**
This study provides a mathematical, mechanistic model of highly pathogenic avian influenza (HPAI) transmission and control in Great Britain during the 2022-23 epidemic. Although adapted from previous studies, the model is fitted to the observed data on infected premises in Great Britain, which is to my knowledge the first time this has been done for this country. As such, this is one of the strengths of this study, which uses advanced fitting method to achieve this aim. The model and methodology are sound and well described, and the code and scripts are available on GitHub which is appreciated and will ease reproducibility. Beyond these methodological aspects, the authors also evaluate how further reducing susceptibility of poultry farms (e.g., through improving biosecurity measures such as cleaning and disinfection or potentially through reactive vaccination) could have impacted the 2022-23 epidemic. Results suggest that major outbreaks could have been avoided with such enhanced control measures, especially if they are implemented in large areas around IPs. This study is timely and can both be used as a reference to model HPAI in other contexts and also to inform policy. I believe it is of interest to the readership of the journal.<br><br>However, before publication, I have a few major comments that need to be addressed, and some minor comments that could contribute to improve the clarity of the manuscript.	We thank the reviewer for their detailed review. We are generally in agreement with their comments and suggestions. A detailed summary is provided below.

Major comments

Reviewer	Response
One of my first comment is that you should make clear everywhere in the text (including abstract) that what you tested is the impact of a reduced susceptibility. You did not model explicitly vaccination or enhanced biosecurity measures, just assumed that those were potential ways of reducing farms susceptibility. You notably mention in the discussion that you could in the future explicitly model vaccination and I agree. Please highlight the differences you would bring to the model if you were to do that (see also my comment below).	We thank the reviewer for this important clarification on how we present our methodology and agree we should make clear that we are not explicitly simulating specific control interventions. As the reviewer notes, instead we characterise the effects of interventions by reducing premises' susceptibility.<br><br>We have adapted our phrasing to clarify that we are only changing the susceptibility. We have made appropriate changes in the abstract, author summary, introduction, methods and discussion to emphasise this point. In the discussion, we have also explained the model adaptations required to simulate potential vaccination interventions.<br><br>The changes are detailed specifically in relation to multiple minor comments below.
You mention in the introduction possible transmission pathways of HPAI between poultry premises, either directly or indirectly. In particular, you discard airborne transmission and you mention that premises-to-premises transmission is likely due to the movement of vehicles. It should be made clearer which transmission mechanisms are captured by your force infection and which ones are not, if any. Also, specify that you do not explicitly account for transmission through vehicle movements (e.g., network model).	We agree with the reviewer about the need to clarify what transmission routes are modelled. We do not discard any particular mechanism and assume all modes will be captured in our transmission kernel approach (even if the precise modes of transmission are not modelled explicitly using different kernels). We note that this is a standard approach used for kernel based infectious disease models, whereby multiple transmission routes (that may include transmission through vehicle movements) are subsumed into a single distance dependent dispersal kernel, with the kernel fitted to observed case reporting data (e.g. Keeling et al. 2001, Tildesley et al. 2006, Probert et al. 2018). This flexible approach allows us to capture the transmission risk to the poultry industry, given that the precise contribution of different transmission mechanisms are unknown.<br><br>In our methods section we have now clarified this and note no network model is involved in our modelling:<br><br>"The local infection component from other poultry premises could reflect a range of different transmission modes, for example: direct transmission by infected poultry; indirect transmission via wild birds as bridging vectors, or; transmission of virus on shared farming equipment or by staff. We also note that cases on premises in close proximity could be indicative of

	an increased presence of H5N1 in local wild bird populations, causing multiple spillover events. Therefore, both components of the infectious pressure may be due to wild bird spillover, with known poultry infections spatially indicating potential higher-risk areas. No transmission routes are excluded in these terms, but likewise none are explicitly modelled, unlike, for example, in models in which a network of vehicle movements were modelled explicitly.”
Also, I understand that in your force of infection you cannot distinguish direct premises-to-premises transmission from spillover from wild birds as they are all intermingled, however I strongly suggest to quantify how many IPs were due to the background infection (first term of the infectious pressure) and how many were due to local infections (second and third terms of the infectious pressure). This would help better understand the results and their interpretation (see comment below).	The reviewer’s understanding is correct that we cannot distinguish direct premises-to-premises transmission in the model from spillover or alternative transmission routes.<br><br>It is certainly possible, in a modelling sense, to quantify which IPs are caused by the background infection term and which are not. This distinction, however, does not provide information on how much infection can be attributed to wildlife as opposed to premises-to-premises transmission (please, see our elaboration on the “local infection component” above).<br><br>As such, we feel that presenting these numbers could be misleading, as we do not want to be misinterpreted that we expect substantial direct between premises transmission events, given that this cannot be established with our model framework. Thus, after substantial reflection, we have decided to omit these numbers. However, for transparency in this document, the proportion of IPs caused by the background term is 27.8% (14.1%–51.6%) across model simulations, with the remainer captured in the “between premises” terms.
Finally, and probably most importantly, when seeing the baseline scenario on figure 3, I am a bit worried: the 200 observed IPs in the data are at the lower end of the 95% prediction interval, which makes me wonder on the quality of the model fit and seems to be in contradiction with Fig 2A. I would expect the predicted number of reported IPs to have a distribution around the observed number of IPs, i.e., the median simulation value of the baseline scenario closer to 200… Please provide explanations for this.	Thank you for this comment. From Figure 2A, we can see the modelled trajectories can replicate the true dynamics, and due to the stochastic and spatial nature of the model, we would not expect all trajectories to look similar. In Figure 2A, large 95% prediction intervals can be seen, where some trajectories incur many more cases. As the reviewer notes, the real outbreak was towards the bottom of the prediction interval. However, as we have established that the model is capable of generating the observed outbreak, we do not believe this to be a serious concern. Of course, the empirical data represent a single stochastic outbreak realisation, and the reported outbreak may have occurred differently in another given year. To reflect on the reviewer’s comment and ensure that we are transparent about this, we have noted this explicitly in the revised manuscript.<br><br>“The data points for the weekly number of premises reported as infected fall within the 95%

	prediction intervals of the model simulations, although generally towards the lower end of these model projections (Figure 2A). However, the closest-matching model trajectories show strong agreement with the data, indicating that the model can successfully replicate the outbreak and provides a good temporal fit.”

Minor comments

Reviewer	Response
**Abstract:** For clarity (see my comment above), change “Our results indicate that enhanced biosecurity measures and/or vaccination” by “Our results indicate that reducing susceptibility (e.g., through enhanced biosecurity measures and/or vaccination)”	We agree that specifying the mechanisms of the model improves the clarity and so have made the suggested change.
**Author summary:** “over the course of a season” -> “over the course of an epidemic season”	We agree with this change.
**Introduction:** Line 13: “hundreds of infected premises” -> I think you could be more precise and provide the actual number of IPs	We agree that this is a reasonable change and so have replace “hundreds of” with “approximately 200”.
On seasonal pattern vs endemic circulation, please explicit when seasonal patterns were observed and when endemic circulation was observed, e.g., was it during the 2022 summer?	We have clarified this instance by explaining that there is a consistent seasonal pattern with a substantial increase in cases over the summer since 2022, indicating possible endemic circulation.<br><br>“… while the seasonal pattern remains, there has been an increased incidence in observed IPs over the summer months, with HPAI detections throughout the summer since 2022.”
Lines 23-26: “The majority of transmission to poultry has generally been attributed to wild ducks, geese, and gulls and in most cases is due to environmental contamination of infected faecal matter in water sources or direct contact with infected carcasses [16].” -> I am not convinced by this sentence, and I did not find supporting evidence for this in reference 16. I am especially struggling to see how direct contact with infected carcasses could represent a major transmission pathway from wild birds to domestic poultry, especially in a western European context. Although I would be more incline to consider contamination of water as a potential transmission route, I don’t know if this has been evidenced in Great Britain or other European countries? I would tend to consider that there would be some safeguards in place to ensure the sanitary status of the water distribution circuits in commercial poultry farming.	We thank the reviewer for this correction. We have changed the reference to be specific to the UK context and have updated as follows, based on *Highly Pathogenic Avian Influenza H5N1 Outbreaks in Great Britain, October 2022 to September 2023:*<br><br>“The majority of transmission to poultry has generally been attributed to Charadriiformes (such as waders, gulls and auks) and Anseriformes (such as ducks and geese) and in most cases this is due to direct or indirect contact, or through contamination of bedding or feed."

Lines 30-31: "Phylogenetic analyses have identified premises-to-premises transmission as being likely for only a few select IPs [21]." -> Please specify that this is true for 2020-2022	We agree that specifying the dates is an improvement and edited the main text accordingly.
Lines 32-33: "Where premises-to-premises transmission does occur, it is likely due to the movement of vehicles or shared equipment between premises [22, 23]." -> Reference [23] is from the Republic of Korea. Although in agreement with the authors' point, studies in other contexts (e.g., France) showed limited role of vehicle movements in premises-to-premises transmission. This should be discussed. Moreover, you never mention again movement of vehicles later in the text.	We agree with the reviewer that we were reductive in claiming premises transmission is likely based on movement of vehicles and equipment. We have updated this sentence to include shared personnel or movement of infected birds. This is in-line with *Highly Pathogenic Avian Influenza H5N1 Outbreaks in Great Britain, October 2022 to September 2023,* which concludes that shared staff, vehicles, egg collection or infected bird transportation are likely sources of infection in these years. We have added this reference.<br><br>"Where premises-to-premises transmission does occur, it is likely due to the movement of vehicles, shared equipment or personnel between premises, or by the transport of infected birds to a new premises."
Lines 33-35: "Airborne transmission between premises is unlikely since evidence suggests that airborne particles containing HPAI virus can only travel very short distances (up to 10 metres) [24]." -> I would suggest to be more precise, as airborne transmission of HPAI in general IMO remains unclear and probably requires further investigation. For instance: "During the 2022-23 season, airborne transmission between premises was unlikely since evidence suggests that airborne particles containing HPAI virus only travelled very short distances (up to 10 metres) [24]."	We agree with the reviewer that specifying the years for this evidence is a useful addition.
Line 37: replace "movement" by "introduction"	We have made this change.
Lines 48-50: Did you include changes in transmission dynamics in your model to account for these housing orders that were not in place at the beginning of the 2022-23 season?	We do not explicitly include housing orders in our model fitting process. We believe that due to the fast-changing and spatially heterogeneous nature of the interventions it would require too many additional parameters to fit all of these changes. However, the impact of the interventions will be captured within the values of the fitted parameters, such as the transmission rate, and these interventions are assumed to have occurred in our baseline scenarios.
Lines 50-52: did you account for the effect of protection and surveillance zones in your model? Were there any reactive culling involved (i.e., culling around infected premises)?	Similarly, we do not explicitly model the effects of the protection and surveillance zones, since our model can capture this in the transmission distance kernel shape. We also did not include any reactive culling since this was very rare in the study period and only when specific direct links were known rather than due to close proximity.
Lines 64-66 and 67-71: I think you should highlight even more in the introduction the fact that this is (at least to my knowledge) the first mechanistic	We thank the reviewer for appreciating the originality of our study and have added the following sentence:

model fitted to HPAI epidemic data in Great Britain and not just a simulation model, which is one of the originalities of your study.	“We believe this is the first mechanistic model fitted to recent HPAI epidemic data in Great Britain.”
**Methods:** Lines 86-87: “We had demographic data from 1 December 2022, which falls within our fitting period.” -> What do you mean “from 1 December 2022”? Do you have temporal information on your demographic data? Later on I understand that you have the data registered on 1 December 2022, is that correct?	The reviewer is correct that we use fixed (non-temporal) demographic data that was registered on 1 December 2022. We have changed this sentence to “We use demographic data that was registered on 1 December…”
Lines 109-110: “all susceptible poultry will be culled unless specific exemption criteria apply” -> what kind of exemption criteria are we talking about? Does this mean that some infected poultry could be left in place?	We have clarified some examples of exemption criteria and added an additional reference to the policy document *Notifiable avian disease control strategy*.<br><br>“(such as some birds from zoos, circuses or pet shops).”
Lines 159-160: “premises, where there is an additional multiplicative scaling parameter γ1 applied to the force of infection from notified premises” -> This has already been said, I would remove it here.	We agree that this repetition could be avoided and have deleted this.
Lines 160-161: “We also consider an exponential kernel in S1 Text.” -> Maybe explicit that this is to see how the shape of your kernel impacts your results.	We thank the reviewer for this addition and have added “to consider how the shape of the kernel impacts our results.”
Line 172: “but there is little specific literature on between-flock latency periods [50].” -> Although I agree, you have some more information in this other literature review (https://doi.org/10.1186/s13567-023-01219-0) on previously used between-flock latency periods that are in line with your assumed value of four days.	We thank the reviewer for this new citation, which we have added to the manuscript alongside the explanation:<br><br>“Four days is in agreement with the between-flock latency period of previous studies.”
Lines 176-177: “This allows for individual differences dependent on the specific premises, but provides an estimate that falls within the typical distribution [50].” -> I would rephrase to avoid confusion, as you did not estimate this parameter. Maybe you could also give the mean of the distribution (in days) to help the reader get a better idea of the duration of the time to notification.	We agree with the reviewer that more clarity is needed here. However, we do in fact fit this parameter for each individual premises, as the given distribution is just our prior on this. We have amended the description as follows:<br><br>“The time in the infected class before notification is fitted for each premises, using a prior of N_j - I_j ~ Gamma(4,2) for the number of days to notification.”<br><br>We also have added the reviewer's suggestion of showing the mean value:<br><br>“…noting the mean value of our prior is eight days”
Line 184: how do you choose the value of the shape of the transmission kernel?	The values were assumed from a previous study and so we have provided an additional citation here, stating:<br><br>“… based on previous studies (Probert et al.).”

Text S1: "We observe that none of these parameters diverge significantly for the prior estimates, but do form smooth distributions that are unique from the priors, indicating that the fitting process has been successful (Figure S4)." -> I am not sure I understand what you mean, and the two first part of the sentence seems a bit contradictory. Were your posterior distributions informed by your data or not? Please clarify.	We agree that this was confusing and have reworded the sentence to show that the data have informed the posterior distributions.<br><br>"We observe that the posterior distributions for all parameters differ from their priors while remaining smooth and well-constrained, indicating that the data have informed the parameter distributions and that the fitting process converged successfully."
Lines 189-195: please better define what you are calling 'occult' infections. In this paragraph, you mention that these are infected but not yet notified, but you do not mention that this is at the end of your simulations (i.e., at the end of the 2022-23 period), i.e., to account for potential missing IPs in your data set at the end of the season.	The reviewer has correctly understood the definition of 'occult infections', but we have adapted our description for clarity.<br><br>"Since HPAI is a notifiable disease, these undetected 'occult' infections will be close to the end of the data period, as while the premises are infectious, they have not been infected for sufficient time to be notified."
On occult infections and time to notification: I do not really understand from your description how that worked. In Text S1, you mention that updating the time of notification and adding/removing occult infections are additional events that represent 5% of the total number of IPs. However, later on, you explain the results on occult infections and time to notification as if they were estimated in your model. Please provide further explanations.	We agree this was unclear. The 5% refers to the potential to add, remove or change the time to notification of 10 IPs per iteration of the MCMC process (since 5% of 200 is 10). Therefore, we have fitted the time to notification and number of occult infections. We have added the following clarification in the SI:<br><br>"We choose the fixed number of additional events to be equal to 5% of the total number of infected premises in the data set. Therefore, we include ten possible additional events per MCMC iteration in the fitting process."
Text S1: "Indeed, we truly observed zero IPs for the time scale of occult infections on 30 September 2023, in agreement with the modelled results." -> I am not sure to understand this sentence. By definition "occult infections" are not (yet) notified and so cannot be observed, how can you be sure that there were no occult infections in real life?	The reviewer is correct that occult infections cannot be observed. However, we now have access to data beyond September 2023, which allows us to conclude that in fact no occult infections occurred at that time.<br><br>"Indeed, using data since September 2023, …"
Line 199: this quite a lot of infected premises at the beginning of the season. Does this mean that there was a persistence of the virus during the summer of 2022?	Yes, we agree with the reviewer that this is a lot of infection, suggesting the virus persisted endemically over the summer season of 2022. We hope we have now clarified this point thanks to the reviewer's previous comments in the introduction.
Lines 201-202: "The numbers were determined from our data set." -> How? In particular, how did you defined them as exposed/infected/notified?	We agree that this needed to be stated. We have clarified this:<br><br>"The numbers were determined from our data set, using the known notification times, where the infection status will vary between simulations, given the distributions for the time to notification."
Line 203: Please provide here or in Text S1 a few lines explaining the grid-based system and how it	We thank the reviewer for asking this question. We do in fact consider each premises separately.

works (e.g., do you model the number of susceptible/exposed/infected/notified/removed farms in each grid cell instead of modelling individual farms?). Also, specify in the main text the size of the grid cells.	We have added the following details to the methods section describing our approach.<br><br>"The algorithm is called the conditional subsample algorithm and, rather than considering each infected premises potentially infecting all other premises sequentially, it reduces the number of calculations required. By dividing the country into large 10 km grid cells, we first assess whether any transmission events occur to any premises within each grid cell, before considering pairwise transmission to premises within that cell. This approach omits many unlikely calculations for transmission over large distances. The algorithm is fully described in Sellman et al."
Line 211: "the potential use of vaccination" -> please specify that you are talking about reactive or ring vaccination, but not preventive vaccination.	We thank the reviewer for pointing this out. We have added:<br><br>"…the potential use of ring vaccination in response to IPs."
Lines 211-213: "All these measures will have the effect in the model of reducing the susceptibility of the poultry that could become infected with HPAI, and so the risk of HPAI incursion." -> "We consider in our model that all these measures will have the effect of reducing the susceptibility of the poultry that could become infected with HPAI, and so the risk of HPAI incursion."	We have made this suggested change.
Line 217: How did you apply 5-10-15 km radiuses if you used 10 km × 10 km grid cells?	We hope that we have clarified the method with the above comment (in response to the reviewer's comment on Line 203). The grid's purpose is to facilitate computational efficiency of model simulations, not to be used to aggregate premises.
Line 218: why did you not test a country-wide scenario? You mention that there were national AIPZ and housing order in place, so why not consider the possibility of national enhanced control?	We wanted to consider an emergency protective response in reaction to IPs in proximity to other premises. While a country-wide response would be simple to implement in the model, we feel that optimising the timing for national responses throughout the period is beyond the scope of our study.
**Results:** Lines 227-228: In addition to this sentence, please add:<br>- A table with some summary statistics (e.g., median or mean and 95% CrI) for the priors and posteriors of the sixteen parameters that were estimated. This would be useful in addition to Figure S4.<br>- One sentence saying that the posterior parameter estimates remain broadly unchanged when considering an exponential kernel and referring to Text S1.	We have added a table of the fitted parameter values to the S1 Text (Table S1) alongside the plotted distributions (Figure S4) and have added a sentence about the similar results for the exponential kernel as requested:<br>"Posterior parameter estimates are described in detail in S1 Text and we note that these estimates remained broadly unchanged when considering an exponential, rather than the results for the Cauchy kernel presented here."
Lines 239-244: From Figure 1 it also seems that most IPs in Scotland occurred late in the season (maybe related to infections in sea bird	Yes, the reviewer is correct that most Scottish IPs occurred towards the end of the season, and the spatially homogeneous background infection

colonies?). Could the observed discrepancy in Scotland also come from the fact that your background infection term in the infectious pressure is temporally but not spatially variable?	term means it is difficult to capture this behaviour. This is discussed in the discussion.
Figure 2A: could you explain why you have so little variability in your simulations between week 39 and week 40? Is this related to the initial conditions?	The reviewer is correct. We have clarified this in the caption.<br><br>"Note that the initial narrow model prediction intervals are due to the initial conditions of infected premises that do not become notified until the second week."
Lines 253-256: see my comments above in the Methods on occult infections.	We have addressed this comment above.
Lines 266 and 278-279: it seems indeed that reducing the susceptibility factor reduces the uncertainty by removing most of the worst-case scenarios and lowering the upper bound of the 95% prediction interval. In contrast, it does not seem to have a big impact on the lower bound of the interval, i.e., there are still dozens or even hundreds of IPs despite the enhanced control measures. How do you explain this? Does the reduced susceptibility impact the background infection pressure and if not, could it be an explanation?	The reviewer is again correct in their understanding. We do not reduce the susceptibility impact from the background infection pressure. We have added:<br><br>"We note that the lower prediction interval is not significantly impacted, because the background infectious pressure term is not impacted by the reduction in susceptibility in our model simulations."
Figure 4: the values of the duration are missing on the y-axis	We thank the reviewer for pointing out this obvious error. We have corrected the labels.
**Discussion:** You mention the potential difficulty of having a homogenous enhanced control and it is definitely relevant. However, I am missing other potential limitations of improved biosecurity, e.g., is this technically and logistically feasible? Is it possible to further improve beyond what is already done and improved through AIPZ and protection/surveillance zones? Do you think poultry farmers still have some room for improvements? Usually, biosecurity measures are already demanding a lot from them, so I wonder how much more it is possible to ask from them.	We agree with the reviewer that additional biosecurity may be difficult to achieve, although is likely possible to some extent. We feel that, as mathematical modellers, it is our place to present the results of the potential impact of improved control, not to speculate on how best to improve control practically. In addition, our framing is for 'enhanced control strategies', which includes vaccination. This presents a clear pathway for enhanced control, since vaccination is not currently in use for HPAI protection in poultry.
Lines 373-377: I agree. Please discuss how considering specifically vaccination could change your results, e.g., there could be a delay before vaccination has an effect (time needed to develop protective immunity) as in reference [33], there could be an effect not only on susceptibility but also on transmissibility…	Thank you for this comment, as it is something we would like to consider. The discussion has been amended to add these details.<br><br>"By modelling vaccination explicitly, we could also consider the impact of reducing the transmissibility of HPAI amongst poultry that were both vaccinated and infected, which would not occur with other enhanced control measures, and would likely lead to even fewer infected IPs. Time delays due to vaccine implementation or vaccination developing protective immunity could also be incorporated."

Reviewer #2:

General comments

**Reviewer**	**Response**
The authors modelled the transmission dynamics between poultry premises across Great Britain between October 2022 and September 2023. They developed an individual-based spatial compartmental model and fitted the demography (spatial coordinates and poultry number) and case data of poultry premises in Great Britain. Using the fitted parameters, they stochastically simulated the epidemics of the season, and projected the impacts of reducing susceptibility by controls, when varying the strength and scale (vicinity of previously infected premises) of control measures. Their main conclusions were that the model captures the temporal and spatial dynamics of the number of affected premises, and that increasing the size of control area radius and control measure strength would be beneficial. The study well showcases using modelling and data to understand the spatial dynamics of avian influenza between farms/poultry premises and control impacts, but there are a few things that need to be addressed.	We thank the reviewer for taking time to review our work. Please find responses to your comments below, along with a description of the changes that we have made to the manuscript to reflect the helpful points raised.

Major comments

**Reviewer**	**Response**
Although the demography data include the number of poultry by species (authors also used this for transmissibility parameter) and I assume the case data have the number of reported cases, the model only fits the number of affected premises, without fitting case number of each premise. Therefore the major conclusions of fitted results and projected impacts are all based on the number or proportion of affected premises. It would be exciting to see if the fitted results can recover the reported cases in Figure 1, and how control measures would impact the number of cases.	Thank you for this excellent point. Poultry premises are not uniformly distributed, and the number of birds varies greatly between premises (shown in Figure S1). Therefore, we have added an additional model validation figure (based on Figure 2) showing the number of birds on the infected premises, rather than the number of IPs only. This new figure has been placed in the supplementary material as Figure S10 and referenced in the main manuscript. The figure shows a similarly strong fit of the simulations to the real data and much more closely matches the data for the East of England.
Regarding the background infection directly caused by spillover from wild birds in the model, this parameter assumes the spillover occurs without spatial heterogeneity as the authors have addressed in Discussion, but also another assumption here is that it assumes the spillover events occur to all farms - another possibility though is that spillovers are limited and cause the initial (few) introductions in a region and the continuing onward transmission is due to transmission among premises. It would be	Thank you for this interesting point. We agree with the reviewer that there may be a small number of incursions of infection from outside Great Britain, with substantial transmission then occurring locally. In principle, our model allows this, since we include two transmission terms: a background infection term and the dispersal term (which could capture, among other transmission routes, transmission directly between premises).<br><br>Of course, in reality the background infection term could (indeed, is likely to) vary both spatially

exciting if authors could model this alternative scenario.<br><br>Related to the last point, the deviations of E England and Scotland simulated results from the data - is it possibly due to that the outbreaks were independent or separate from other regions? For example, a spillover event in Scotland that happens in summer (according to Reference 14) when the seasonal forcing of background infection can't fully capture (Figure S9); and for E England, perhaps there weren't so much background infection as the parameter represents, and were mostly local transmission between poultry premises as they start with many affected premises already. Perhaps the authors can simulate a few scenarios to test the possibilities; or, drawing data of wild bird cases to inform the parameter of background infection (spillover from wild birds).	and temporally. The reviewer is correct that one possibility is that there is a greater background infection risk in summer in Scotland than our temporal (but not spatial) function suggests, and similarly there may be a lower background infection risk in East England. However, we contend that being able to model such scenarios accurately would require detailed additional data (e.g., both presence and absence data for infections in wild birds) and, correspondingly, a more complicated mathematical model. We do not have access to such data, and therefore we think that a better approach is to use a simple model (as we have done) to replicate the broad pattern of infections in the poultry industry across the UK.<br><br>Nonetheless, we note in the Discussion of the revised manuscript that adding more detail in the background infection term, including spatial heterogeneity, is a key area for future work; although this would require additional data, it may allow the spatial distribution of infections across Great Britain to be captured more accurately by the model, at the cost of additional model parameter values to fit or assume.

Minor comments

Reviewer	Response
Line 123: "susceptible to infection" should be "susceptible to exposed"	We have changed this to "susceptible to HPAI infection." We mean a susceptible bird capable of being infected, not one moving from the susceptible class to the exposed class.
Line 127: "according" should be removed	We have now corrected the sentence to read as "...according to the..."
Figure 4: the values for duration of control (on the axis) are missing	We thank the reviewer for pointing out this obvious error. We have corrected the labels.
Line 26-28, a suggestion on describing the species-level difference in susceptibility: "Chickens and turkeys infected with HPAI typically show more severe symptoms or higher mortality compared to ducks and geese, although the latter may have similar levels of viral shedding without showing as much symptom or mortality. This difference, however, may be less obvious for some genotypes of the circulating H5N1 clade 2.3.4.4b, as they are particularly well adapted to ducks." The citations here can add this article of experimental infections of two genotypes of clade 2.3.4.4b of various species including chickens, ducks and geese.<br><br>Bordes L et al. 2024 Experimental infection of chickens, Pekin ducks, Eurasian wigeons and	We thank the reviewer for this more detailed explanation and have amended the paragraph as suggested, including the reference.

Barnacle geese with two recent highly pathogenic avian influenza H5N1 clade 2.3.4.4b viruses, Emerging Microbes & Infections, 13:1, 2399970, http://dx.doi.org/10.1080/22221751.2024.2399970	

References

1. Keeling MJ, Woolhouse ME, Shaw DJ, Matthews L, Chase-Topping M, Haydon DT, Cornell SJ, Kappey J, Wilesmith J, Grenfell BT. Dynamics of the 2001 UK foot and mouth epidemic: stochastic dispersal in a heterogeneous landscape. Science. 2001 Oct 26;294(5543):813-7.
2. Tildesley MJ, Savill NJ, Shaw DJ, Deardon R, Brooks SP, Woolhouse ME, Grenfell BT, Keeling MJ. Optimal reactive vaccination strategies for a foot-and-mouth outbreak in the UK. Nature. 2006 Mar 2;440(7080):83-6.
3. Probert WJM, Jewell CP, Werkman M, Fonnesbeck CJ, Goto Y, Runge MC, et al. (2018) Real-time decision-making during emergency disease outbreaks. PLoS Comput Biol 14(7): e1006202.
4. Sellman S, Tsao K, Tildesley MJ, Brommesson P, Webb CT, Wennergren U, Keeling MJ, Lindström T. Need for speed: An optimized gridding approach for spatially explicit disease simulations. PLoS computational biology. 2018 Apr 6;14(4):e1006086.
