# Supplementary material for: A modelling assessment of the impact of control measures on highly pathogenic avian influenza transmission in poultry in Great Britain
Source: PLoS Comput Biol. 2026 Jan 5;22(1):e1013874. doi: 10.1371/journal.pcbi.1013874 (PMC12810912; doi:10.1371/journal.pcbi.1013874)
Supplement: Attachment — Submitted filename: reviewer_response_2.pdf [file pcbi.1013874.s003.pdf]

Dear Prof. Blyuss and Prof. Flegg,

Thank you for the opportunity to revise our manuscript following the useful comments from the reviewers. The reviewers have provided valuable feedback, and we have fully addressed their comments, particularly adding a more detailed justification for our approach (including areas that could be adapted in future work) to the discussion section. We hope that our manuscript is now acceptable for publication in PLOS Computational Biology.

A full response to the reviewers' comments is detailed below.

Yours sincerely,

Christopher Davis

University of Warwick

(on behalf of all co-authors)

Reviewer #1:

General comments:

**Reviewer**	**Response**
The authors made a good effort of clarification and accounted for most of both reviewers' comments. I believe the manuscript has been improved. I only have a few minor comments. Lines below refer to the version with tracked changes.	We thank the reviewer for taking the time to provide further comments on our manuscript.
The authors chose not to provide the number of IPs caused by the background term versus the number of IPs caused by the local terms. While I understand the reason behind their decisions, I still think that this is important for the interpretation of the alternative scenarios with reduced susceptibility. Indeed, this reduced susceptibility is only applied to the local terms (which should be made clearer lines 240-261) and not to the background terms, which explains some aspects of the results presented in Figure 3. Although I agree that this does not provide information on the contribution of wildlife as opposed to premises-to-premises transmission, I believe that this result could still be provided if worded carefully so as not to be misinterpreted by the reader. This substantial contribution of the background term should also be added in the explanation lines 340-342.	On further consideration, we believe the reviewer makes a valuable point and have now included the proportion of IPs generated by the background term. This does indeed help to explain the results of Figure 3.<br><br>To the start of the results section, we have added:<br><br>"In these model simulations, 27.8% (95% prediction interval: 14.1%–51.6%) of the IPs arise due to the background term epsilon, with the remainder due to the local infection components."<br><br>We have updated lines 340–342:<br><br>"We note that the lower prediction interval is not impacted substantially, because we keep the background infectious pressure term epsilon constant across all premises and only reduce the susceptibility due to the local infection component."<br><br>And added within lines 240–261:

	“In practice, this scales our beta_ij term (which includes the premises susceptibility component) by a given proportion, termed the susceptibility factor, while we leave the background infection term epsilon unchanged.
Regarding spatio-temporal changes of the interventions (e.g., housing orders), you mention that these will be captured within fitted parameter values. Although I agree, these will be captured on average over the entire study region and the entire study period. Do you think that explicitly accounting for spatio-temporal interventions could have improved the spatio-temporal fit of your model, e.g., reduce the overestimated number of outbreaks in some of your simulations? If yes, you could briefly discuss this in your manuscript.	The reviewer raises a valid point that by fitting a time and spatially varying intervention term, we could have seen an improvement in the model fitting. We have added this comment to the discussion:<br><br>“We could also explicitly account for the impact of biosecurity and housing orders within the model fitting process by introducing more parameters, rather than assuming these effects are captured within the baseline parameters. This could improve the model fit by enabling the model simulations to include additional spatial heterogeneity compared to our current results.”

Minor comments:

Reviewer	Response
Line 51: I missed this during the first round of review, but what do you mean by “improved fencing”? I can see how fencing of pastures can be effective for domestic mammals (cows, pigs…) to reduce contacts with wildlife, but I am having trouble seeing how this can work for birds.	Thank you for the question. We mention that improved fencing could be one of a number of measures used to increase biosecurity, which, as the reviewer states, is to reduce contact with wildlife. In particular, we reference a study in South Korea by Yoo et al., where improved fencing was found to be effective in commercial duck farms at reducing infection by limiting contact with wild species, although it had a limited impact for layer chickens.<br><br>We have added the clarification:<br><br>“… and improved fencing to reduce contact between poultry and wild bird species.”
Line 137: maybe you could add “exposed to HPAI infection E (i.e., infected but not yet able to transmit infection)”	We agree with the reviewer that this change improves the clarity of the manuscript.
Line 139-141: there is a potential confusion between the time of infection, the time at which premises became exposed E, and the time at which premises became I. For clarity, maybe the I compartment should be renamed as infectious instead of infected, and make sure that there is no confusion between the time of infection (i.e., when premises became exposed) and the onset of infectiousness (i.e., when premises moved from E to I).	We agree there could be confusion in the difference between our infection classes, so have adopted the suggestions of the reviewer. We have changed the suggested lines below and updated mentions of the “infected class” to the “infectious class” throughout the manuscript.<br><br>“…infectious and able to transmit infection I, notified as infected but still infectious N, and removed by culling R. For each premises i, we denote E_i as the time of infection and so when the premises becomes exposed. This similarly applies to the time of the onset of infectiousness

	(I_i), the time of notification (N_i), and the time of culling (R_i)."
Line 144-145: is the background time-varying term only capturing infections caused by spillover from wild birds, or could it also implicitly capture other transmission routes, e.g., long-range transmission by vehicles movements that are not captured by the local components?	Thank you for this point. Long range transmission events are still possible (with low probability) in the local component, due to the decaying transmission kernel as distance increases. Therefore, we interpret the background term as only capturing spillover from wild birds.
Lines 232-239: why is your addition appearing in red and crossed out? This is useful and should appear in the final manuscript.	Thank you for pointing out this mistake. This was meant to be included in the revised manuscript; we have addressed this.
Lines 340-342: maybe you should explain why the background term is not impacted by reduced susceptibility. I guess it is because it would apply to all farms in the country, whereas you are interested in local improvements around IPs?	The reviewer is correct. We agree that this should be clearly explained (alongside the General comment #2) to help the reader understand our model. We have changed this sentence:<br><br>"We note that the lower prediction interval is not impacted substantially, because we keep the background infectious pressure term epsilon constant across all premises and only reduce the susceptibility due to the local infection component.
Table S1: "parameter" (an "e" was missing)	We have corrected this typo.

Reviewer #2:

Major comments

**Reviewer**	**Response**
Regarding my second major comment, it seems to be not completely understood and I would like to clarify. By "another possibility though is that spillovers are limited and cause the initial (few) introductions in a region and the continuing onward transmission is due to transmission among premises.", I meant that the initial introductions could be limited in some premises in a spatial region. Since the background infection is modelled as a constant in the model for all the premises, the model can't account for this spatial heterogeneity. The authors mentioned they have discussed this limitation in the article and I believe they were referring to this paragraph: "In designing our model, we have made the simplifying assumption that background infectious pressure from wild bird spillover into poultry premises is spatially uniform across Great Britain. This could be challenged by incorporating spatial information on wild bird habitats and detected cases into the ϵ term in the model equations (Equation 2). Alternatively, known environmental sources of infection or reported	We thank the reviewer for their insightful comment. The reviewer has highlighted the correct paragraph of the discussion that explains that habitat data or pseudo-premises for wild bird infections could potentially improve the spatial model fit. However, this would be difficult to implement because of biases in the available data sets. These wild bird case data for Great Britain rely on passive surveillance and thus will be biased by clear mortality events (such as mass die-offs of seabird colonies). There will also be substantial under-reporting due to the lack of systematic national testing, and the absence of any false positives in these data sets makes it difficult to assess the extent of under-reporting. This is why in the previous response we referred to a lack of data.<br><br>To reflect the reviewer's comment in our manuscript, we have now more clearly reasoned why we have not used these data in the discussion:

wild bird cases could be added as pseudo-premises to the model to include additional transmission sources. However, we have shown that we are able to achieve a remarkably good match to the real-world data for the 2022–23 season, given the lack of this information in the model (Figure 2)." However, this explanation is different from authors' response to my comment where the authors stressed the limitation is due to lack of data.<br><br>Reported wild bird cases could be accessed from Food and Agricultural Organization of the United Nations (FAO) Empres-i and World Animal Health Information System database provided by the World Organization for Animal Health. It seems that authors could incorporating thses data into ϵ or adding wild bird cases as pseudo-premises, as they suggested themselves to address the spatial heterogeneity of introduction or background infection.<br><br>If these approaches are both limited by some other issues, I suggest the authors add other limitations or edit the relevant text in the discussion to provide a more sufficient explanation.	“However, since reported wild bird case numbers rely on the passive surveillance of found dead birds, there will likely be substantial underreporting and biases in the locations where dead birds are more likely to be found. Therefore, incorporating these data could skew the model results.
Nontheless, I believe my other suggestion of simulating senarios of different levels of background infection would still work, without modifying the model - since the model is individual premise based, the authors could have varying background infection terms for premises in different region. For example, to test if the deviation of Scotland data from the model is caused by a separate introduction in the summer and continuing local transmisison within Scotland, the authors can simulate four senarios: 1. specifiy a different ϵ for all premises in Scotland, and separate these premises from the rest (assuming there is no transmission between Scotland and all other premises); 2. only specify a different ϵ for all premises in Scotland; 3. only separate Scotland premises from the rest; 4. the originally modelled scenario, and see which result would better reflect the data. Again, if these tests would not be possibly made using the current model or data, I sugges the authors to include relevant limitations and future work in the article.	This is a very helpful suggestion and is a clear goal for a future publication. In fact, we have already begun to consider how importations of infection may vary across Great Britain, and we will use the reviewer’s suggestion to inform this ongoing work. We intend to use our current premises infection data to fit a background infection term epsilon (and peak timing and peak shape parameters nu) separately for each region of Great Britain (for Scotland, England, and Wales in the first instance). This would therefore use our existing data to improve our spatial model fit (albeit at the cost of additional model parameters to estimate).<br><br>For this paper, we believe there is value in the relative simplicity of the current modelling approach, given the good quality of the model fit without a more complicated design, and so we have not included this here. However, we have used the reviewer’s suggestion to add to our discussion and highlight this future work:<br><br>“Alternatively, using only the current data sets, future work could investigate fitting the underlying model parameters of the background infection term (epsilon_0, nu_0 and nu_1) separately for each region of Great Britain. This could resolve issues with the spatial model fit, at the cost of additional model parameters to estimate.”
